# Gestational age and birth weight variations in young children with language impairment at an early communication intervention clinic

**DOI:** 10.4102/sajcd.v65i1.584

**Published:** 2018-09-17

**Authors:** Lauren C. Fouché, Alta Kritzinger, Talita le Roux

**Affiliations:** 1 Department of Speech-Language Pathology and Audiology, University of Pretoria, South Africa

## Abstract

**Background:**

South Africa presents with high preterm birth (PTB) and low birth weight (LBW) rates (14.17%). Numerous conditions characterised by language impairment are associated with LBW and/or PTB. Speech-language therapists may fail to identify older children whose language impairment may have originated from LBW and/or PTB.

**Objective:**

To describe the frequency of LBW and/or PTB, in comparison with full-term birth, and associated conditions in children at an early communication intervention (ECI) clinic.

**Methods:**

Retrospective data of 530 children aged 3–74 months were analysed, with 91.9% presenting with language impairment.

**Results:**

Almost 40% had LBW and/or PTB, and late PTB was the largest category. Factors associated with LBW and/or PTB were prenatal risks, including small-for-gestational age, perinatal risks, including caesarean section, and primary developmental conditions. Secondary language impairment was prevalent, associated with genetic conditions and global developmental delay.

**Conclusion:**

The frequency of LBW and/or PTB was unexpectedly high, drawing attention to the origins of language impairment in almost 40% of the caseload at the ECI clinic.

## Introduction

As a low-to-middle-income country, South Africa presents with preterm birth (PTB) (< 37 weeks gestational age) and low birth weight (LBW) (< 2500 g) rates as high as 14.17%, as opposed to 7% in high-income countries (Feresu, Harlow, & Woelk, 2015; Howson, Kinney, & Lawn, 2012; Pattinson, 2013). Apart from a high mortality risk and neonatal illness, the neurodevelopmental concerns within this population of children are diverse and can be long term (Allen, 2008). Severe developmental disabilities associated with LBW and/or PTB include cerebral palsy (CP), sensory impairments of vision and hearing, mental disability and seizure disorder (Allen, 2008; Van de Weijer-Bergsma, Wijnroks, & Jongmans, 2008). These disabilities may all be associated with secondary language impairment. In addition, neurodevelopmental functions, such as attention, cognition, executive functioning, emergent literacy, sensory processing, gross and fine motor skills, communication and language, as well as feeding and swallowing, may be affected in children with LBW and/or PTB, and associated with less severe clusters of impairments and disorders (Allen, 2008; Howson et al., 2012; Mathisen, Carey, & Brien, 2012). Language impairment in children born preterm and with LBW may also co-occur secondary to various genetic syndromes or congenital conditions. One of these conditions is Foetal Alcohol Spectrum Disorder (FASD), a congenital disorder affecting 6% of the South African population (Foundation for Alcohol Related Research [FARR], 2016). Children born with FASD are small-for-gestational age, which is associated with intrauterine growth restriction and often also accompanied by PTB (Peranich, Reynolds, O’Brien, Bosch, & Cranfill, 2010). Low birth weight and/or preterm birth has recently been identified as a risk for autism spectrum disorder (ASD) (Kihara & Nakamura, 2015). Infants born with syndromic cleft lip and/or palate (CLP) are at risk for lower birth weight of up to 600 g less than unaffected infants (Nyarko, Lopez-Camelo, Castilla, & Wehby, 2013). Similarly, infants who are exposed to the human immunodeficiency virus (HIV) are at risk for very LBW, atypical length and head circumference, and neurodevelopmental deficits and feeding difficulties associated with HIV-encephalopathy (Kihara & Nakamura, 2015). It has also been found that LBW could be associated with Down syndrome (Nyarko et al., 2013). Low birth weight and/or preterm birth are thus recognised as potential causes of language impairment and swallowing disorder in young children with a wide variety of neurodevelopmental conditions (Mathisen et al., 2012).

In addition to the numerous neurodevelopmental sequelae that may result from LBW and/or PTB, it is also necessary to consider the wide array of underlying biological, medical and environmental interactional factors significantly associated with LBW and/or PTB. Genetic influence, race and ethnicity, very young and advanced maternal age, *in vitro* fertilisation, multiple pregnancies, maternal history of PTB, maternal infections and chronic conditions such as diabetes and high blood pressure, smoking, alcohol and substance abuse, reduced spacing between births, poor antenatal care and nutrition, induced or caesarian birth, high levels of outdoor air pollution, poverty, minority status and certain migrant subgroups have been shown to be associated with LBW and/or PTB (Barrett, 2014; Howson et al., 2012; Johansson & Cnattigius, 2010; Organisation for Economic Co-operation and Development [OECD]/World Health Organization [WHO], 2016; Urquia et al., 2010). Globally, the risks for LBW and/or PTB are increasing, with preventative efforts not yet effective (Editorial, 2016; Howson et al., 2012). It is clear that LBW and/or PTB is a complex condition in children requiring current knowledge and in-depth understanding of all its implications.

The terms LBW and PTB are distinct, yet closely related indicators and are recommended to be used in combination to indicate a new born infant’s immaturity. Intrauterine growth restriction may cause misclassification of gestational age and over-representation in studies when only birth weight is used (Johansson & Cnattigius, 2010). Infants with LBW and/or PTB do not have an established risk for delayed development but may represent an at-risk population with neurodevelopmental disabilities or disorders, often presenting as complex co-occurring conditions within one child (Rossetti, 2001).

Speech-language therapists and other health professionals may fail to identify disorders related to LBW and/or PTB in their caseloads of older children. The detail of case histories of older children and the significance of late PTB may be overlooked. There may also be a perception that infants with LBW and/or PTB outgrow early language delay (Van Niekerk, Kirsten, Nel, & Blaauw, 2014). Research is required to better understand the prevalence of and co-occurring conditions associated with language difficulties in preschool children who were born preterm and with LBW.

While preterm infants are expected to achieve normal growth and weight between the ages of 2 and 3 years (Rasmussen, Wong, Correa, Gambrell, & Friedman, 2006), recent studies indicate that many developmental sequelae may persist throughout life (American Psychiatric Association [APA], 2013; Bailey & Sokol, 2008; Kihara & Nakamura, 2015; Nyarko et al., 2013; Van de Weijer-Bergsma et al., 2008). Language impairment in children with LBW and/or PTB involve deficits in receptive and expressive components, grammar, vocabulary and articulation (Feldman, Lee, Yeatman, & Yeom, 2012; Schults, Tulviste, & Haan, 2013; Wolke, Samara, Bracewell, & Marlow, 2008). The language difficulties may persist throughout primary school, a period during which language development should stabilise and mature (Boyer et al., 2014; Wolke et al., 2008). It is not clear from these studies whether children with LBW and/or PTB present mostly with primary language impairment or language impairment secondary to conditions associated with LBW and/or PTB.

Preterm birth and LBW is therefore considered as a complex condition that may originate from different causal factors, and may be associated with a wide array of outcomes, affecting the child in various degrees of severity and persistence. Since it is a prevalent condition in South Africa and other low-to-middle income countries (Pattinson, 2013), a description of the frequency of LBW and/or PTB, co-occurring conditions and developmental characteristics of children with language impairment may create an increased awareness in speech-language therapists and health care professionals managing these children. The purpose of this study was, therefore, to describe the frequency of LBW and/or PTB, in comparison to being born with normal birth weight (NBW) and full term (FT), as well as associated conditions in children at an early communication intervention (ECI) clinic.

## Methods

### Study sample

A comparative, retrospective design was used. The study was conducted at an ECI university-based clinic. The clinic provides assessment and intervention services to young children and their families, who visit the clinic owing to concerns regarding the child’s communication and language development. On average, 50 new clients are annually assessed at the clinic. A uniform assessment protocol, including the *Rossetti Infant-Toddler Language Scale* (Rossetti, 2006), was used for all play-based communication assessments, and informed consent was obtained from parents prior to each assessment.

All children who attended the ECI clinic between 2003 and 2015, and of whom complete datasets were available, were included in the study. The datasets equated to 530 participants, which consisted of preschool children (mean = 28.47 months, mode = 13 months, SD = 14.66 months, range = 3–74 months), with a gender distribution of 333 (63%) males and 197 (37%) females. The male gender bias in the sample is a known risk factor in children with language impairment (Korpilahti, Kaljonen, & Jansson-Verkasalo, 2016). Table 1 describes the family and participant demographics of the study sample.

**TABLE 1:** Demographical characteristics of study sample.

Demographics	%	*n*	Mean	SD	Mode	Range (minimum–maximum)
Mother’s age at birth of child	-	-	29.45 years	5.07 years	30 years	17–52 years
**Ethnicity**					
White people	77.0	406	-	-	-	-
African people	19.0	101	-	-	-	-
Asian people	3.60	19	-	-	-	-
Mixed race people	0.40	2	-	-	-	-
**Language exposure of child (*N* = 520)**					
Monolingual	67.1	349†	-	-	-	-
Multilingual	32.9	171†	-	-	-	-
**Home language (*N* = 528)**					
Afrikaans	59.3	313†	-	-	-	-
English	22.2	117†	-	-	-	-
African languages (Northern Sotho, Setswana, etc.)	17.6	93†	-	-	-	-
Other (German, French, etc.)	0.90	5†	-	-	-	-
**Day-care of child (*N* = 506)**					
Stays at home with parent	35.2	178†	-	-	-	-
Attends preschool (half-day)	23.7	120†	-	-	-	-
Attends crèche (full-day)	18.0	91†	-	-	-	-
Stays at home with caregiver other than direct family member	15.6	79†	-	-	-	-
Stays with family member	7.50	38†	-	-	-	-
**Health sector (*N* = 527)**					
Private health care	77.0	406†	-	-	-	-
Public health care	23.0	121†	-	-	-	-
**Residential setting (*N* = 530)**					
Urban	94.9	503	-	-	-	-
Rural	5.10	27	-	-	-	-
**Highest educational qualification of mother (*N* = 511)**					
Tertiary qualification	52.7	264†	-	-	-	-
Grade 12 (Matric)	42.7	218†	-	-	-	-
≤ Grade 11	5.60	29†	-	-	-	-

† , Owing to incomplete patient files, results were based on available data for each variable.

As seen in Table 1, the study sample was diverse but consisted mainly of white people (77%), Afrikaans speaking (59.3%) children who stayed at home with a parent during the day (23.7%) and were exposed to only one language (67.1%). The majority of the sample originated from an urban residential setting (94.9%), made use of private healthcare services (77%), with just more than half (52.7%) of the caregivers having completed tertiary education. The use of private health care and the high education level of mothers suggest that the sample included mostly middle-income families, and as a result, this study sample is not representative of the South African population. The characteristics of the sample may relate to the middle-income urban geographical location of the university where the clinic operates from.

### Data collection procedures and material

The patient register of the ECI clinic was reviewed in order to identify children who were assessed between 2003 and 2015. The clinical files of the children with complete data were drawn from the filing cabinets and then reviewed retrospectively. An electronic database was developed to organise and capture the data in a consistent format. Data collected included demographical and medical information, case history questionnaires containing documented risk factors and communication assessment results.

### Data analysis

STATA statistical software package (version 21) was used to analyse the data. Data were divided into two distinct groups: the LBW and/or PTB group, which included all children who were born preterm, or LBW or were born preterm with LBW; and the NBW and/ or FT group, which included children who were FT or post-term, with normal or high birth weight. For this distinction, the WHO classification system for PTB and LBW was used (Howson et al., 2012; Reidy et al., 2013). Preterm birth was categorised into three gestational ages: extremely preterm (< 28 weeks gestational age), very PTB (28 – < 32 weeks gestational age) and moderate to late preterm (32–37 weeks gestational age); and LBW was categorised as: LBW (< 2500 g), very LBW (< 1500 g) and extremely LBW (< 1000 g) (Howson et al., 2012; Reidy et al., 2013). The ECI clinic where data were collected provides intervention services to children who are within the pre-lingual period, as well as those who are busy acquiring language. The population receiving ECI services at the clinic, therefore, includes children with communication delay, but the term language impairment as suggested by Owens (2014) was used to refer to the multiple aspects of the children’s communication and language difficulties. The degrees of language impairment were used as recommended by Rossetti (2006), where mild refers to a 3–6 month delay, moderate refers to a 6–12 month delay and severe refers to a 15 month delay or more. A criterion-referenced developmental assessment scale (Anderson, Fowler, & Nelson, 1978) was used to determine the children’s developmental status regarding socio-emotional, perceptual-cognitive, play, gross and fine motor, and self-help skills.

In this study, primary language impairment refers to language impairment as the only relevant factor, whereas secondary language impairment is used when a child presented with a condition other than his or her language difficulties, which may have language impairment as a secondary outcome. Global developmental delay was used when a child does not meet his or her developmental milestones in several areas of intellectual functioning at the appropriate age (APA, 2013). Genetic or congenital conditions and ASD occurring in the sample were diagnosed by an independent medical specialist.

Descriptive statistics were calculated for all variables, including means, standard deviations and proportions. Student’s *t*-tests were used to determine significant differences between the two study groups. The Chi-Squared test (Fischer’s exact) was used to identify significant associations between variables. Associations between variables were investigated for the two groups to determine if there were significant differences between children with LBW and/or PTB, and children born FT with NBW. A significance level of *p* ≤ 0.05 was deemed significant. Owing to the retrospective nature of the study, patient files were not all complete for every variable considered. Results were, therefore, based on the available data for each variable.

## Ethical consideration

Institutional ethical clearance was obtained for the study before data collection was initiated (protocol number 12142078-GW20160314HS).

## Results

### Frequency of low birth weight and preterm birth

Table 2 depicts the mean gestational age and birth weight for the two groups, respectively.

**TABLE 2:** Frequency of low birth weight and preterm birth.

Characteristic	Low birth weight and/or preterm birth				Normal birth weight and full-term or post-term birth			
	*n*	%	SD	Range	*n*	%	SD	Range
**Gestational age**								
Number of children in data set (*n* = 528; 2 missing values)	209	39.4	-	-	319	60.6	-	-
Mean gestational age in weeks	35.0	-	3.4	-	39.3	-	1.1	-
Weeks (minimum–maximum)	-	-	-	26–37	-	-	-	38–44
**Birth weight**								
Number of children in data set (*n* = 521; 9 missing values)	206	39.5	-	-	315	60.5	-	-
Mean birth weight in grams	2303.3	-	720.3	-	3269.3	-	402.2	-
Grams (minimum–maximum)	-	-	-	620–2500	-	-	-	2501–5100

According to Table 2, 209 (39.4%) of the children were in the LBW and/or PTB group, and 319 (60.6%) in the NBW and/or FT group (*n* = 528). The average gestational age for the LBW and/or PTB group was 35 weeks, which is considered late preterm (32–37 weeks), whereas the average for the NBW and/or FT group was 39.3 weeks, which is FT (38–41 weeks). The average birth weight for the LBW and/or PTB group was 2303.3 g, which corresponds with infants born late preterm (Stolt et al., 2016), and 3269.3 g for the NBW and/or FT group. The distribution of birth weight and gestational age across the study sample can be seen in Table 3.

**TABLE 3:** Distribution of birth weight and gestation age (*N* = 530).

Categories	%	*n*
**Birth weight category**†		
< 1000 g	2.60	14
< 1500 g	3.60	19
< 2500 g	21.5	114
≥ 2500 g	72.3	383
**Gestation age category**†		
Extremely preterm (< 28 weeks)	2.50	13
Very preterm (28–32 weeks)	3.80	20
Late preterm (32–37 weeks)	27.0	143
Full term (38–41 weeks)	64.7	343
Post term (< 42 weeks)	2.10	11

g, grams.

† , Data on birth weight category were available for all children, however, the exact birth weight was unknown for nine children (as indicated in Table 2);

† , data on gestation age category were available for all children; however, the exact gestation age was unknown for two children (as indicated in Table 2).

Table 3 shows that NBW (72.3%) and FT (64.7%) combined with post-term birth (2.1%) were the most prevalent groups in the sample. The second largest groups were 21.5% of the study sample with a birth weight of between 1500 g and 2500 g (LBW), and 27% with a gestational age within the late preterm range. This corresponds with the average birth weight and gestational age of the LBW and/or PTB group (Table 2), highlighting the prevalence of children born late preterm and with LBW but not in the very LBW category in this sample. The difference in percentages between birth weight and PTB, which is expected to correspond, is owing to infants being born small-for-gestational age (below the 10th percentile for gestation), thus their birth weight is lower than the expected weight for gestational age. Just more than 6% of the study sample had a birth weight of less than 1500 g, and were born before 32 weeks’ gestational age, representing the small group of very LBW and very PTB in the sample. The majority of this sample with language concerns presented with birth weights above 1500 g (LBW) and were born later during the gestation period (after 32 weeks).

### Language impairment

Table 4 indicates the distribution of the degrees of language delay across the entire study sample, as well as distribution thereof between the two study groups. Although all children in the sample attended the ECI clinic owing to concerns regarding their communication and language development, only 91.86% of the entire sample were found to present with primary or secondary language impairment after assessment at the clinic. Presence of language impairment for a few children could not be determined owing to a very young age, and the remaining were found to have typical language development. The reason why 33 out of 528 children (6.25%) of the sample did not present with language delay could be related to parents requesting an assessment but no delay was found. Ten children (1.89%) were infants under three months of age who presented with Down syndrome or another established risk condition but could not be scored on the *Rossetti Infant-Toddler Language Scale* (Rossetti, 2006) as the instrument requires a delay of more than three months to be classified as language delay.

**TABLE 4:** Degrees of language delay across study sample.

Degree of language delay	Distribution across entire study sample (*N* = 528†)		Low birth weight and/or preterm birth group (*N* = 209)		Normal birth weight and full-term or post-term birth group (*N* = 319)	
	%	*n*	%	*n*	%	*n*
None	6.250	33	3.30	7	8.2	26
Mild	21.02	111	23.5	49	19.4	62
Moderate	21.78	115	23.9	50	20.4	65
Severe	49.05	259	45.9	96	51.1	163
Cannot be determined owing to age under three months	1.890	10	3.30	7	0.9	3

† , Data on the degree of language delay were available for the entire sample, except for two children.

According to Table 4, almost half (49.1%) of the entire study sample had severe language delay. Within the LBW and/or PTB group, 93.4% of children had language delay. Differences between the LBW and/or PTB group and the NBW and/or FT group were minimal in all degrees of language impairment, with differences of no more than 5.2% between groups. This indicates that LBW and/or PTB children within this sample did not present with more severe language impairment than their FT peers with language impairment.

### Primary developmental conditions

The primary developmental conditions of the children in this study sample can be seen in Table 5. The *Established Risk Categories* according to Rossetti (2001) were used to categorise the different conditions found in the sample.

**TABLE 5:** Primary developmental conditions associated with preterm birth and low birth weight.

Primary developmental conditions (*N* = 530): primary condition child presents with at time of assessment	Low birth weight and/or preterm birth (*N* = 209)		Normal birth weight and full-term or post-term birth (*N* = 321)		*p*-value
	*n*	%	*n*	%	
Genetic conditions: Pierre Robin sequence Down syndrome Velo-cardio-facial syndrome	39.0	18.7	28	8.7	< 0.001**
Global developmental delay	30.0	14.4	28	8.7	0.04**
ASD	16.0	7.7	54	16.8	0.002**
Non-syndromic cleft lip and/or palate	45.0	21.5	90	28.0	0.09*
Primary language impairment	58.0	27.8	86	26.8	0.81
Neurological conditions: seizure disorder cerebral palsy microcephaly unspecified brain disorder	12.0	5.7	15	4.7	0.58
Hearing disorders: including auditory neuropathy spectrum disorder conductive hearing loss and sensorineural hearing loss	3.0	1.4	6	1.9	1.00
FASD	3.0	1.4	1	0.3	0.31
Feeding difficulties	2.0	1.0	1	0.3	0.57
STORCH infection: HIV-exposure cytomegalovirus)	1.0	0.5	2	0.6	1.00
Recurrent otitis media	0.0	0.0	4	1.2	0.16
Visual impairment	0.0	0.0	3	0.9	0.28
Post meningitis syndrome	0.0	0.0	2	0.6	0.52
Metabolic disorder	0.0	0.0	1	0.3	1.00

ASD, autism spectrum disorder; FASD, Foetal Alcohol Spectrum Disorder; STORCH, foetal infections that may cause congenital malformations: syphilis, toxoplasmosis, other infections, rubella, cytomegalovirus infection and herpes simplex; HIV, human immunodeficiency virus.

* , Marginal significance (0.05 < *p* < 0.1);

** , significant (*p* < 0.05).

According to Table 5, a wide array of primary developmental conditions and accompanied language impairment occurred in both study groups. Statistically significant differences were found between the two groups for genetic conditions, global developmental delay, ASD and non-syndromic CLP. Some of the conditions occurred more in the LBW and/or PTB group, while some conditions were statistically more prevalent in the NBW and/or FT group. Genetic conditions and global developmental delay were strongly more significant in children with LBW/PTB (18.7%) than those born NBW and/or FT (8.7%). Global developmental delay was statistically more significant in the LBW and/or PTB group. Cleft lip and/or palate occurred more frequently in children born NBW and/or FT, but only marginally significantly. This finding shows that LBW and/or PTB did not occur more in children with non-syndromic CLP, but when accompanied by a genetic condition, such as Pierre Robin sequence and Velo-cardio-facial syndrome, LBW and/or PTB can be expected. Autism spectrum disorder was highly associated with children born NBW/FT, but not in the LBW and/or PTB group. However, LBW and/or PTB occurred in 22.5% of the 70 children with ASD, presenting much higher than the South African LBW and/or PTB rate of 14.17%.

Primary language impairment without any associated conditions was equally represented in both groups and was also the most prevalent developmental condition for both groups. No significant difference in the frequency of primary language impairment was found between the two groups, indicating that primary language impairment was not increased in the LBW and/or PTB sample. Within the sample of children with LBW and/or PTB, 27.8% presented with primary language impairment, and 68.9% presented with secondary language impairment. As already indicated in Table 4, almost all children with LBW and/or PTB assessed at the clinic presented with some form of language impairment, but language impairment was not more severe in the LBW and/or PTB group.

Preterm birth and LBW did not occur significantly more in any of the other primary developmental conditions. The conditions occurred too rarely in the sample to show any significant differences.

### Risk factors associated with low birth weight and preterm birth

Table 6 depicts which risk factors were significantly associated with the LBW and/or PTB group. The different risk factors considered under prenatal, postnatal and family risks are supplied in Table 7. Factors showing no association with LBW and/or PTB in the sample were gender, ethnicity, family risk for language impairment, feeding difficulties at the time of assessment, family risks (such as low education level of parents), use of public or private medical services, mono- or multilingual language exposure and child attendance of day-care.

**TABLE 6:** Associations between preterm birth and/or low birth weight and other risk factors.

Other risk factors associated with LBW and or PTB	*p*-value
Small-for-gestational age	< 0.001**
Maternal prenatal risks	< 0.001**
Multiple pregnancy	< 0.001**
Postnatal risks: feeding difficulties long NICU stay	< 0.001**
Primary developmental condition at time of assessment (see Table 4)	< 0.001**
Caesarean section delivery	0.002**
Primary and secondary language impairment	0.03**
Mild to moderate language impairment	0.03**
History of otitis media	0.05*
Low Apgar score at birth	0.08*
Surgical procedures early in life: cleft repair tonsillectomy heart surgery, etc.	0.06*

NICU, neonatal intensive care unit; LBW, low birth weight; PTB, preterm birth.

* , Marginal significance (0.05 < *p* < 0.1);

** , significant (*p* < 0.05).

**TABLE 7:** Prenatal, postnatal and environmental risks associated with low birth weight and preterm birth.

Risk factors associated with LBW and PTB	Low birth weight and/or preterm birth		Normal birth weight and full-term or post-term birth		*p*-value
	*n*	%	*n*	%	
**Maternal prenatal risks (*N* = 526†)**	207	-	319	-	< 0.001
Mother older than 37 years	9	4.3	11	3.4	
Multiple pregnancy	13	6.2	6	1.9	
High blood pressure, Pre-eclampsia, HELLP syndrome	26	12.6	13	4.1	
Medical conditions and treatment	82	39.6	89	27.9	
Smoking, alcohol and drugs	25	12.1	23	7.2	
Mental health conditions	9	4.3	3	0.9	
Placenta problems	15	7.2	2	0.6	
No risk	68	32.9	195	61.3	
**Postnatal risks (*N* = 530)**	209	-	321	-	<0.001
Feeding difficulties	66	31.6	58	18.1	
NICU stay ≥ 5 days	113	54.1	52	16.2	
NICU stay < 5 days	13	6.2	15	4.7	
No risk	70	33.5	217	67.6	
**Family risks (*N* = 530)**	209	-	321	-	0.61
Poverty suspected	2	1.0	12	3.7	
Abuse or neglect	3	1.4	1	0.3	
Foster care	5	2.4	5	1.6	
Low education levels of parents	2	1.0	4	1.2	
Little or poor stimulation from caregivers	11	5.3	13	4.1	
Adolescent mother	1	0.5	2	0.6	
Postnatal hospitalisation of infant	3	1.4	2	0.6	
Alcoholic caregivers	0	0.0	1	0.3	
No risk	195	93.3	294	91.6	

HELLP, haemolysis, elevated liver enzymes, and low platelet count; NICU, neonatal intensive care unit; LBW, low birth weight; PTB, high preterm birth.

† , Data unavailable for four children.

According to Table 6, the most significant risk factors associated with LBW and/or PTB in this study sample were small-for-gestational age, maternal prenatal risks, multiple pregnancy, postnatal risks, the primary developmental condition (genetic conditions and global developmental delay), caesarean section delivery and language impairment, specifically in the mild to moderate category. Infants born small-for-gestational age are usually LBW, hence the association with the LBW and/or PTB group. Prenatal risk factors, such as medical conditions and treatment during pregnancy, high blood pressure, pre-eclampsia, multiple pregnancy, smoking and alcohol use during pregnancy, pose known risks for preterm labour and delivery of an infant, and were, therefore, significantly associated with LBW and/or PTB (Nyarko et al., 2013; Reidy et al., 2013; Rossetti, 2001; Wardlaw, Blanc, Zupan, & Ahman, 2004). Postnatal risks, such as length of stay in the neonatal intensive care unit (NICU) and feeding difficulties, were associated with LBW and/or PTB, as 126 (60.3%) of the children in the sample were admitted to the NICU. The primary developmental conditions associated with LBW and/or PTB were genetic conditions, and global developmental delay, as also indicated in Table 3. Elective caesarean section deliveries are known to contribute to late PTB (Howson et al., 2012). Caesarean births in South Africa have been reported to constitute 21% of births in general, and 43.1% of births among white women (Biswas, Su, & Mattar, 2013; Department of Health, 2007). Although the male gender was predominant in this study sample, there was no significant association found with LBW and/or PTB.

## Discussion

Study results indicated that almost 40% of children assessed at an ECI clinic were born preterm with LBW. The sample consisted mostly of young preschool children with a mean age of 28.47 months, and 13 months as the mode. Almost all children in the study sample (91.9%) had language impairment. This high frequency occurrence is linked to the clinic’s typical service users, that is, young children with communication and language concerns. The majority of the preterm children in the sample, 143 out of 176 (81.25%), were late preterm (35 weeks), with LBW (2303.3 g). It is known that late PTB represents the largest group of children with LBW and/or PTB, occurring five times more than children born before 32 weeks’ gestational age, or 80% of the population (Saigal & Doyle, 2008; Vohr, 2010). Because of late preterm infants being almost similar in size and weight to their FT peers, they are often treated in the same manner as an FT infant by both parents and medical professionals (Engle, Tomashek, & Wallman, 2007). These infants are physiologically and metabolically immature, placing them at high risk for medical complications and infant mortality (Engle et al., 2007). Within this study sample, 95.8% of late PTB children presented with language impairment. Although the results represent data from a single ECI clinic from a majority middle-income group, the distribution of LBW/PTB categories was similar to those reported by Vohr (2010). The sample comprised participants with access to private health insurance; however, their study population was of a higher educational background (Vohr, 2010).

The current study also highlights the many associated conditions and high prevalence of secondary language impairment in the children with late PTB. It appears that research typically focuses on the language and communication outcomes of very preterm and extremely preterm children (Nosarti, Murray, & Hack, 2010; Rayco-Solon, Fulford, & Prentice, 2005; Sansavini et al., 2015). Late PTB children can, therefore, easily be overlooked, as the focus of research is often on the population at greater risk for severe disability. Seizure disorder, CP and other neurological conditions occurred rarely in the current sample and were not more prevalent in the LBW and/or PTB group than in the NBW and/or FT group.

Conditions known to be associated with LBW and/or PTB were genetic conditions and global developmental delay. Genetic conditions, such as Down syndrome which was the most common genetic condition found in the study sample (45/530; 8.4%), are linked to LBW and/or PTB (Rasmussen et al., 2006). Velo-cardio-facial syndrome, also known as 22q11.2 deletion syndrome, occurred in four (0.75%) children in the sample. Pierre Robin sequence is a genetic condition and occurred in 11 children within this sample (2.1%), with an incidence varying between 1 in 8500 and 1 in 14 000 live births (Rathé et al., 2015). The condition starts developing during embryology, and is caused by a sequence of events caused by micrognathia, resulting in posterior placement of the tongue, which in turn prevents complete closure of the palate (de Buys Roessingh, Herzog, Cherpillod, Trichet-Zbinden, & Hohlfeld, 2008). Global developmental delay, a diagnosis given to children under five years who have not met several developmental milestones at the appropriate age, and who cannot yet be reliably assessed for intellectual disability (APA, 2013), was frequently found in the sample (58/530; 10.9%). It appears that global developmental delay is the most frequently occurring developmental condition in children with LB and/or /PTB, and that it predicts language development (Rushe, 2010). Autism spectrum disorder has been identified as a risk for LBW and/or PTB (Kihara & Nakamura, 2015). In this study, 70 (13.2%) children had ASD, and 16 (22.5%) of these children had LBW and/or PTB. This number is considerably higher than the national LBW rate in South Africa, therefore, this study confirms the risk for LBW and/or PTB in children with ASD. All these associated conditions add to the complexity of children with LBW and/or PTB and can easily mask their low gestational age and birth weight. Therefore, most children with LBW and/or PTB within this study sample presented with multiple risk factors, language impairment and often another developmental disorder as well.

Primary language impairment did not occur more in the LBW and/or PTB group (27.8%) than in the NBW and/or FT group (26.8%). Rushe (2010) found that, when children with cognitive disability are excluded from comparisons, the prevalence of language impairment between children born preterm and those born at term remains consistent. This perspective suggests that language impairment in children with LBW and/or PTB occurs secondary to their cognitive disability. In contrast, Schults et al. (2013) found that more children with LBW and/or PTB present with primary language impairment, with poorer expressive and receptive language, than their FT counterparts. Further research is required to better understand language impairment in children with LBW and/or PTB.

Factors found to be most significantly associated with PTB and LBW in this study were small-for-gestational age, maternal prenatal risks, caesarean section delivery and postnatal risks. Maternal prenatal risks were found to be significantly associated with LBW and/or PTB. Factors such as medical conditions and treatment during pregnancy; mental health conditions; placental problems such as pre-eclampsia; multiple pregnancy; and smoking, alcohol and drug use during pregnancy are known risks for LBW and/or PTB (Allen, 2008; Nyarko et al., 2013; Reidy et al., 2013; Sansavini et al., 2015; Stolt et al., 2016). It is also known that, for various reasons, multiple pregnancy often results in PTB before 32 weeks gestational age (Allen, 2008; Zhu, Tao, Hao, Sun, & Jiang, 2010). Postnatal risk factors were significantly associated with LBW and/or PTB in this study. In the study sample, postnatal risk factors included feeding difficulties and a period in a NICU. Infants born preterm often present with feeding difficulties as their neurological systems are still underdeveloped (Mathisen et al., 2012; Nosarti et al., 2010; Rayco-Solon et al., 2005).

## Conclusion

The frequency of LBW and/or PTB was almost 40% in the study sample of children with language impairment. In this predominantly late LBW and/or PTB sample, secondary language impairment was prevalent and found to be associated with genetic conditions and global developmental delay. The finding concurs with some studies suggesting that primary language impairment is not more prevalent among children with LBW and/or PTB than among those born NBW and/or FT, but rather occurs as a secondary impairment. The large sample size of the study strengthens the reliability of the findings. Limitations of the study include the sample size being limited to a single ECI clinic with a predominantly middle-to-high income population within South Africa, thus not offering insight applicable to the wider population of children with language impairment in South Africa. Furthermore, the LBW and/or PTB group were compared to FT children with language impairment, not with typically developing children. Further research in the prevalence of language impairment among preterm infants within the South African population, and the distinction between primary and secondary language impairment within this population, may provide further insight into the impact that PTB and/ or LBW has on language development.

Children with LBW and/or PTB can be identified early, as they enter the health care system upon their untimely birth. With a known risk for language impairment and subsequent risk for learning difficulties, timely ECI is essential. The LBW and/or PTB population in South Africa and elsewhere is a growing health concern requiring attention and public awareness. Young children with LBW and/or PTB and their mothers should ultimately benefit from the National Integrated Early Childhood Development System, as ‘many disabilities are preventable or could have their severity limited if pregnant women, infants and young children received access to early quality screening, preventative and rehabilitative care’ (Republic of South Africa [RSA], 2015:24).
