# Corrigendum: Gestational age and birth weight variations in young children with language impairment at an early communication intervention clinic

**DOI:** 10.4102/sajcd.v66i1.622

**Published:** 2019-09-12

**Authors:** Lauren C. Fouché, Alta Kritzinger, Talita Le Roux

**Affiliations:** 1 Department of Speech-Language Pathology and Audiology, University of Pretoria, South Africa

In the initial published version of this article, the competing interest statement was omitted and the acknowledgment statement was incorrect. The statement is hereby provided and updated as follows: ‘The authors declare no potential conflicts of interest with respect to the research, authorship and/or publication of this article.’

The acknowledgments paragraph is hereby updated as follows: ‘L.C.F. thanks Prof. A. Kritzinger and Dr T. Le Roux for their continued support, guidance and valuable input into this article. She also thanks her family and friends, who stood by her through the writing process, lending an ear to listen as she phrased and rephrased sections. She also appreciates all the support she had during her journey.’

These corrections do not alter the study’s findings of significance or overall interpretation of the study results. The author apologises for any inconvenience caused.

